# Experimental phasing using zinc anomalous scattering

**DOI:** 10.1107/S0907444912024420

**Published:** 2012-08-18

**Authors:** Sun-Shin Cha, Young Jun An, Chang-Sook Jeong, Min-Kyu Kim, Sung-Gyu Lee, Kwang-Hoon Lee, Byung-Ha Oh

**Affiliations:** a Marine Biotechnology Research Center, Korea Institute of Ocean Science and Technology, Ansan 426-744, Republic of Korea; b Department of Marine Biotechnology, University of Science and Technology, Daejeon 305-333, Republic of Korea; c Department of Biological Sciences, KAIST Institute for the Biocentury, Korea Advanced Institute of Science and Technology, Daejeon 305-701, Republic of Korea

**Keywords:** zinc anomalous scattering, phasing, Zn derivatization

## Abstract

Zinc is a suitable metal for anomalous dispersion phasing methods in protein crystallography. Structure determination using zinc anomalous scattering has been almost exclusively limited to proteins with intrinsically bound zinc(s). Here, it is reported that multiple zinc ions can easily be charged onto the surface of proteins with no intrinsic zinc-binding site by using zinc-containing solutions. Zn derivatization of protein surfaces appears to be a largely unnoticed but promising method of protein structure determination.

## Abstract 2

The surface of proteins can be charged with zinc ions and the anomalous signals from these zinc ions can be used for structure determination of proteins.

## 1. Introduction

Experimental phasing is essential to solve protein structures which cannot be determined by molecular replacement. The use of tunable synchrotron radiation has led to the development of multiwavelength anomalous dispersion (MAD) and single-wavelength anomalous dispersion (SAD), powerful phasing methods that are based on the anomalous scattering of certain atoms. Selenium is the most commonly used anomalous scatterer for MAD/SAD because selenomethionine and selenocysteine can be incorporated into recombinant proteins. However, selenium-based MAD or SAD is not applicable to natural proteins and is hardly applicable to recombinant proteins with no methionine or cysteine. In addition, the crystallization of selenium-labelled proteins is sometimes unsuccessful despite the successful crystallization of the corresponding native proteins. In these cases, the introduction of anomalous scatterers such as heavy atoms and halide ions into crystals by soaking is an alternative. Heavy-atom derivatives are usually prepared by soaking multiple crystals in numerous heavy-atom compound solutions containing gold, platinum, mercury or lead for days to weeks, while a quick (less than 1 min) soak of crystals in cryosolutions containing bromides or iodides allows the incorporation of these ions (Dauter & Dauter, 1999 ▶; Dauter *et al.*, 2000 ▶).

Zinc is the second most abundant metal in cells and serves as a cofactor for diverse enzymes and regulatory proteins or contributes to the structural integrity of proteins. It is estimated that about 5–10% of all proteins predicted from the genomes of all three domains of life are zinc-binding proteins (Andreini *et al.*, 2006 ▶). Zinc is an ideal anomalous scatterer for MAD/SAD phasing considering that its strong anomalous signal (*f*″ = 3.9 electrons at its *K* edge) is comparable to that of Se and its absorption edge (λ = 1.284 Å) falls within the normal energy range of macromolecular crystallography beamlines. Crystal structures of many proteins with intrinsically bound zinc have been successfully determined using zinc anomalous scattering. Nevertheless, zinc has rarely been used to prepare heavy-atom derivatives and no zinc compound is included in commercially available heavy-atom screening kits, which seems to be a consequence of the general notion that specific metal-binding sites are a prerequisite for zinc binding. Here, we show that the surface of proteins can be charged with zinc ions and that the anomalous signals from these zinc ions can be used for structure determination of proteins.

## 2. Methods

### 2.1. Protein preparation and crystallization

The gene for CMY-10 was chemically synthesized and subsequent subcloning and purification were performed as described previously (Lee *et al.*, 2004 ▶). Crystals of CMY-10 were grown in a precipitant solution consisting of 18% polyethylene glycol 8000, 0.1 *M* sodium cacodylate pH 6.5, 0.2 *M* zinc acetate dehydrate using the microbatch crystallization method.

The *TON_0340* gene of *Thermococcus onnurineus* NA1 was cloned into pET22b-CPD 10H, an in-house-modified form of pET22b (Novagen), to express a protein fused to His_10_-tagged CPD (cysteinyl protease domain) at the C-terminus (Shen *et al.*, 2009 ▶). The fusion protein was expressed in *Escherichia coli* BL21 (DE3) RIPL strain (Novagen) at 310 K. Bacterial lysates were prepared by sonication in buffer *A* composed of 20 m*M* Tris–HCl pH 7.5, 100 m*M* NaCl, 5 m*M* β-mercaptoethanol. Cleared lysates isolated by centrifugation were loaded onto a column packed with HisPur Cobalt Resin (Thermo) and washed with buffer *A* containing 10 m*M* imidazole. On-gel auto-cleavage of His_10_-tagged CPD was performed by incubating the resin with buffer *A* containing 100 m*M* phytate for 2 h at room temperature, which activates the protease activity of CPD. TON_0340 was eluted with buffer *A* and further purified with a HiTrap Q HP column (GE Healthcare). For crystallization, the final sample was concentrated to 9 mg ml^−1^ in a buffer solution composed of 20 m*M* Tris–HCl pH 7.5, 100 m*M* NaCl, 1 m*M* dithiothreitol. Crystals were obtained by the hanging-drop vapour-diffusion method using a precipitant solution consisting of 5%(*v*/*v*) 2-propanol, 100 m*M* sodium acetate pH 5.0, 350 m*M* zinc acetate at 295 K.

Hen egg-white lysozyme was purchased from BIO Basic Inc. and was used without further purification. Lysozyme crystals were grown at 295 K using the microbatch crystallization method. Small drops composed of 1 µl protein solution (20–50 mg ml^−1^) and an equal volume of a precipitant solution consisting of 1 *M* NaCl, 0.1 *M* citric acid pH 4.0 were pipetted under a layer of a 1:1 mixture of silicon oil and paraffin oil in 72-well HLA plates (Nunc).

### 2.2. Data collection

A 2.1 Å resolution MAD data set for CMY-10 was collected at wavelengths of 1.2825 Å (peak), 1.2828 Å (inflection point) and 1.1700 Å (high-energy remote) using an ADSC Quantum 270 CCD on the microfocus beamline PF-17A at the Photon Factory, Japan. For each data set, 180 diffraction images with a 1° oscillation width were collected with the crystal-to-detector distance set to 180 mm (Table 1 ▶).

A 2.3 Å resolution SAD data set for TON_0340 was collected at the zinc absorption peak using an ADSC Quantum 315 CCD on beamline 4A of Pohang Light Source, Republic of Korea (Table 1 ▶). In order to avoid spot overlaps arising from the long *c* axis of the unit cell, we used a 90°-bent metal pin to align the *c* axis along the spindle axis and collected 200 images of 1° oscillation with the crystal-to-detector distance set to 300 mm (covering 200° of oscillation) that are free of interference from the metal pin (Table 1 ▶).

A 1.8 Å resolution SAD data set for hen egg-white lysozyme was collected at a wavelength of 1.2829 Å using an ADSC Quantum 270 CCD on the microfocus beamline PF-17A at the Photon Factory, Japan. A total of 180 frames of 1° oscillation were collected with the crystal-to-detector distance set to 176 mm (Table 1 ▶).

### 2.3. Data processing and phasing

Diffraction data were processed and scaled using *DENZO* and *SCALEPACK* from the *HKL*-2000 program suite (Otwinowski & Minor, 1997 ▶). Experimental phasing of CMY-10 and lysozyme was performed with the *AutoSol* program (Terwilliger *et al.*, 2009 ▶) in the *PHENIX* suite (Adams *et al.*, 2010 ▶), which is an experimental phasing pipeline that combines *HySS* (*Hybrid Substructure Search*; Grosse-Kunstleve & Adams, 2003 ▶) for finding heavy-atom sites, *Phaser* (McCoy *et al.*, 2007 ▶) or *SOLVE* (Terwilliger, 2002 ▶) for calculating experimental phases and *RESOLVE* (Terwilliger, 2002 ▶) for density modification and model building. Experimental phasing of TON_0340 was performed with *autoSHARP* (Vonrhein *et al.*, 2007 ▶), an automatic structure-solution system that includes the heavy-atom refinement and phasing program *SHARP* (de La Fortelle & Bricogne, 1997 ▶), the density-modification program *SOLOMON* (Abrahams & Leslie, 1996 ▶) and the *ARP*/*wARP* package (Perrakis *et al.*, 1999 ▶) for automated model building and refinement. The auto-built models from the phasing programs were completed using *Coot* (Emsley & Cowtan, 2004 ▶) and refinement was performed with a maximum-likelihood algorithm implemented in *CNS* (Brünger *et al.*, 1998 ▶).

## 3. Results and discussion

### 3.1. Observation of zinc binding to the protein surface

We encountered a paper describing zinc binding to a protein without an intrinsic zinc-binding site, the crystals of which grew in a precipitant solution containing zinc acetate (Axelrod *et al.*, 2010 ▶). According to the crystal structure that was determined based on selenium anomalous scattering (PDB entry 3h50), four zinc ions were bound to surface-exposed aspartate, glutamate and histidine residues. The presence of zinc ions on the protein surface was reminiscent of the crystal structure of CMY-10 (PDB entry 1zkj) determined previously (Kim *et al.*, 2006 ▶). The crystals of this protein also grew in the presence of zinc acetate (Lee *et al.*, 2004 ▶) and its crystal structure contained zinc ions tightly associated with the imidazole ring of histidine, the carboxyl group of aspartate/glutamate or the amide group of asparagine/glutamine on the protein surface. In the two crystal structures most zinc ions bound to histidine residues, indicating that histidine is the preferred residue for zinc coordination on the protein surface. The zinc ions were coordinated in a tetrahedral geometry, except for one that was bound to asparagine with an octahedral geometry. Interestingly, a single amino-acid residue was sufficient to bind a zinc ion because water or acetate molecules also participated in the coordination.

### 3.2. Structure determination of CMY-10 using zinc anomalous scattering

**Figure 1:**
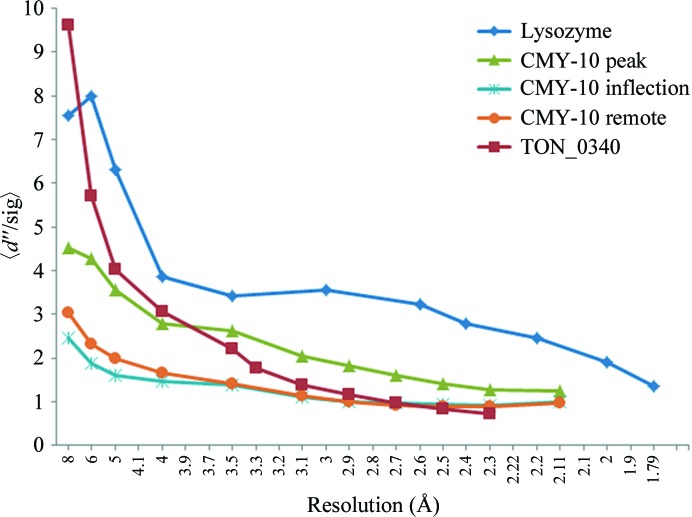
The 〈*d*″/sig〉 plot from *SHELXC* as a function of resolution.

**Figure 2:**
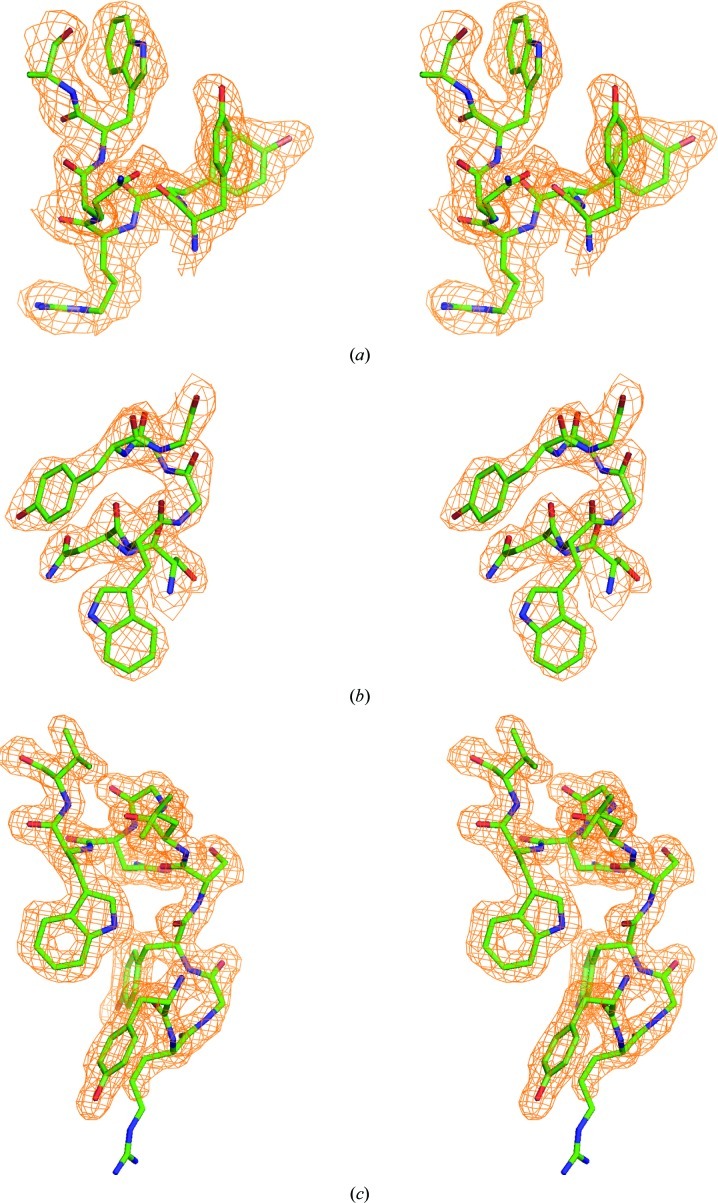
Stereoviews of representative portions of the experimental electron-density maps contoured at 1σ for CMY-10 (*a*), TON_0340 (*b*) and lysozyme (*c*).

We tested whether anomalous signal from the zinc ions bound to the surface of CMY-10 might be sufficient for phase determination. A 2.1 Å resolution MAD data set (Table 1 ▶) was collected at three wavelengths from a crystal that was cooled in a cryostream at 100 K after briefly being immersed in a cryoprotectant solution consisting of 15% glucose, 18%(*w*/*v*) polyethylene glycol 8000, 0.1 *M* sodium cacodylate pH 6.5, 0.2 *M* zinc acetate dehydrate. The CMY-10 crystals, which contained one molecule per asymmetric unit, belonged to the monoclinic space group *P*2_1_, with unit-cell parameters *a* = 49.6, *b* = 59.2, *c* = 63.5 Å, β = 103.7°. Anomalous signal, which was evaluated using 〈*d*″/sig〉, a good indicator of the strength of the anomalous signal, was present to 2.1 Å resolution in all three data sets and was strongest for the peak data set, as expected (Fig. 1 ▶). The *AutoSol* program identified 13 zinc sites, with occupancies in the range 0.19–0.99, and produced a phase set with a figure of merit (FOM) of 0.45. The phases were further improved by density modification, with a final FOM of 0.62. The experimental electron-density map was very clear (Fig. 2 ▶) and a model with *R*_work_/*R*_free_ of 0.2501/0.2784 was automatically built. These results indicate that surface-bound zinc ions can successfully be used for phase determination by the MAD method.

### 3.3. Structure determination of TON_0340 using zinc anomalous scattering

Recently, we obtained crystals of TON_0340 in a precipitant solution containing zinc acetate. Based on our confidence in zinc derivatization and phase determination, we collected a 2.3 Å resolution SAD data set (Table 1 ▶) from a crystal that was cooled in a cryostream at 100 K after briefly being immersed in a cryoprotectant solution consisting of 20% glycerol, 5%(*v*/*v*) 2-propanol, 100 m*M* sodium acetate pH 5.0, 350 m*M* zinc acetate. The TON_0340 crystals, which contained six molecules per asymmetric unit, belonged to the tetragonal space group *P*4_3_2_1_2, with unit-cell parameters *a* = *b* = 107.44, *c* = 355.03 Å. According to the plot of 〈*d*″/sig〉 *versus* resolution, zinc anomalous signal was present, although it decreased drastically at high resolution (Fig. 1 ▶). A total of 66 zinc ions, with occupancies of 0.08–1.00, were identified in the asymmetric unit and the resulting phasing set, characterized by an FOM of 0.33, yielded a clearly interpretable electron-density map (Fig. 2 ▶) leading to an auto-built model with *R*_work_/*R*_free_ of 0.27/0.31. Just like the other cases described above, zinc ions were associated with aspartates, glutamates and histidines on the protein surface. Additionally, three zinc ions bound to a cluster of six acidic residues were located in a putative active-site cleft.

### 3.4. Structure determination of lysozyme using zinc anomalous scattering

In the two cases described above, zinc ions were present in the crystallization solutions. We tested whether the surface of a protein can be charged with zinc ions by soaking protein crystals in a solution containing zinc ions. Crystals of hen egg-white lysozyme were first obtained in a precipitant solution composed of 1 *M* NaCl, 0.1 *M* citrate pH 4.0. The lysozyme crystals were then transferred into a solution consisting of 50 m*M* zinc acetate, 1 *M* NaCl, 0.1 *M* MES pH 6.5, 25% ethylene glycol. We changed the pH from 4.0 to 6.5 to deprotonate histidine, which appears to be the most preferred residue to associate with zinc ions. After 10 min soaking, a lysozyme crystal was mounted and the crystal was flash-cooled at 100 K using a cryostream cooler. A fluorescence scan was performed to locate the Zn *K* edge and a 1.8 Å resolution SAD data set was collected at a wavelength of 1.2829 Å (Table 1 ▶). The lysozyme crystal, which contained one molecule per asymmetric unit, belonged to the tetragonal space group *P*4_3_2_1_2, with unit-cell parameters *a* = *b* = 79.19, *c* = 36.82 Å. The anomalous signal was high over the entire resolution range (Fig. 1 ▶).

**Figure 3:**
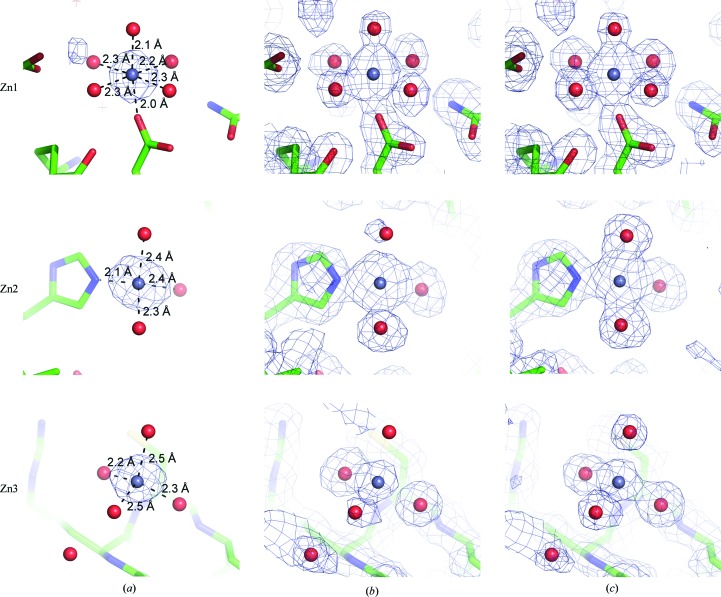
Anomalous Fourier maps at the 5σ level (*a*), experimental electron-density maps after density modification (*b*) and final 2*F*_o_ − *F*_c_ maps (*c*) at the 1σ level superposed onto zinc-binding sites in the final model of lysozyme. Zinc ions and water molecules are represented by blue and red spheres, respectively.

The experimental phase information resulting from the SAD phasing was of high quality; the FOMs before and after density modification were 0.42 and 0.64, respectively. The electron-density map calculated from the experimental phases was readily interpretable (Fig. 2 ▶) and a nearly complete model with *R*_work_/*R*_free_ of 0.1856/0.2323 was automatically built. The root-mean-square deviation for all atoms between zinc-bound lysozyme and zinc-free lysozyme (PDB entry 2lyz; Diamond, 1974 ▶) was only 0.85 Å, indicating that zinc binding had no effect on the structure. Although 21 zinc ions were identified by the phasing program, three zinc ions (Zn1, Zn2 and Zn3) were finally modelled (Table 1 ▶). Zn1 was coordinated by Asp52 and three water molecules in a tetrahedral geometry, whereas Zn2 was coordinated by His15 and five water molecules in an octahedral geometry (Fig. 3 ▶). In the case of Zn3, the zinc ion interacted with four water molecules in a tetrahedral geometry and two of the coordinating water molecules were engaged in weak hydrogen bonds to backbone N atoms (Fig. 3 ▶). The weak association of Zn3 was reflected in its *B* factor (56.86 Å^2^), which was higher than those of Zn1 (31.92 Å^2^) and Zn2 (38.63 Å^2^). The successful zinc SAD phasing suggests that zinc derivatization of protein surfaces by crystal soaking is a method of choice for *de novo* structure determination of proteins for which crystals grow in the absence of zinc ions.

### 3.5. A survey of the Protein Data Bank

We observed that zinc ions derived from crystallization solutions bind to surface-exposed residues. To ascertain the generality of this observation, we searched the Protein Data Bank for proteins for which crystals were grown in solutions containing zinc acetate. The search was stopped after the collection of 43 cases (see Supplementary Material^1^) because all the cases were consistent with our observations. Without exception, zinc ions are associated with surface residues such as histidine, aspartate, glutamate, asparagine or glutamine. Among the collected cases, we found that three structures were solved by Zn MAD phasing [PDB entries 1fd9 (Riboldi-Tunnicliffe *et al.*, 2001 ▶), 2ch9 (Schüttelkopf *et al.*, 2006 ▶) and 3cjj (Koch *et al.*, 2010 ▶)]. The three proteins are not zinc-binding proteins, but the phasing method was tried because zinc ions were essential for crystallization. There are two, four and five zinc ions in the 1fd9, 3cjj and 2ch9 structures, respectively, and their occupancies range from 0.2 to 1. While other protein structures were determined by molecular replacement or selenium-based MAD/SAD phasing, our experimental demonstrations suggest that their structures could have been determined by exploiting the zinc anomalous signal. A survey of the crystallization conditions of the 43 cases showed that the concentration of zinc acetate ranged from 3 to 300 m*M*, indicating that zinc-bound crystals can be prepared at diverse zinc concentrations. The buffer pH range of 4.5–8.3 revealed by this survey might indicate the limit of zinc-binding conditions since zinc-binding residues are protonated at low pH and zinc in solution is precipitated at high pH.

In conclusion, we demonstrated that protein crystals grown in the presence of zinc ions or soaked in a zinc-containing solution are easily charged with multiple zinc ions on the protein surface and that they are suitable for zinc SAD/MAD phasing. A phasing effectiveness of 570 amino acids per fully occupied Zn atom was shown experimentally and simulated MAD phasing suggested that one Zn site is able to provide adequate experimental phase information for as many as 1100 amino acids (Meyer *et al.*, 2006 ▶). Considering the high phasing effectiveness and multiple zinc-binding sites, we believe that zinc derivatization of the protein surface is a largely unnoticed but promising method for phase determination of protein crystals.

## Supplementary Material

PDB reference: zinc-bound lysozyme, 4dt3

PDB reference: TON_0340, 4fc5

Supplementary material file. DOI: 10.1107/S0907444912024420/wd5183sup1.pdf

## Figures and Tables

**Table 1:** Data-collection, refinement and phasing statistics Values in parentheses are for the outer shell.

	CMY-10			TON_0340	Lysozyme
Protein	Peak	Inflection	Remote	SAD	SAD
Data collection					
Space group	*P*2_1_	*P*2_1_	*P*2_1_	*P*4_3_2_1_2	*P*4_3_2_1_2
Wavelength (Å)	1.2825	1.2828	1.1700	1.2822	1.2829
Unit-cell parameters (Å, °)	*a* = 49.59, *b* = 50.15, *c* = 62.53, β = 103.66			*a* = *b* = 107.44, *c* = 355.03	*a* = *b* = 79.10, *c* = 36.82
Resolution (Å)	50–2.10 (2.18–2.10)	50–2.10 (2.18–2.10)	50–2.10 (2.18–2.10)	50–2.30 (2.34–2.30)	50–1.80 (1.85–1.80)
Completeness (%)	97.4 (90.9)	99.8 (99.2)	99.7 (99.1)	99.6 (99.2)	99.9 (100)
*R*_merge_ † (%)	9.5 (29.5)	11.6 (60.7)	10.9 (55.3)	12.3 (48.7)	7.2 (21.8)
〈*I*/σ(*I*)〉	21.9 (3.8)	14.5 (1.8)	14.0 (1.8)	21.3 (3.5)	73.7 (16.4)
Multiplicity	4.7 (3.2)	3.4 (3.0)	3.4 (3.2)	12.7 (8.2)	13.4 (12.9)
〈*d*″/sig〉 of each shell‡	4.53, 4.28, 3.55, 2.78, 2.63, 2.06, 1.84, 1.62, 1.41, 1.27, 1.24	2.45, 1.88, 1.60, 1.48, 1.38, 1.11, 1.01, 0.97, 0.95, 0.92, 0.99	3.04, 2.33, 1.98, 1.65, 1.42, 1.15, 1.01, 0.93, 0.88, 0.90, 0.97	9.60, 5.71, 4.02, 3.06, 2.21, 1.77, 1.40, 1.17, 0.97, 0.85, 0.74	7.54, 7.98, 6.31, 3.85, 3.41, 3.55, 3.24, 2.80, 2.45, 1.90, 1.37
Phasing statistics					
No. of Zn sites finally modelled	12			53	3
Site occupancies§	0.99, 0.86, 0.81, 0.79, 0.64, 0.62, 0.59, 0.53, 0.45, 0.44, 0.40, 0.33			0.35–1.00	0.66, 0.49, 0.48
FOM§					
Before DM	0.45			0.33	0.42
After DM	0.62			0.55	0.62
Model–map CC§	0.81			0.84	0.89
Refinement statistics					
Resolution range (Å)	50–2.10			50–2.30	50–1.80
No. of reflections	37619			87763	20533
No. of atoms					
Protein	2652			12042	995
Chloride ions					8
Ethylene glycol					1
Zinc ions	12			53	3
Acetates	24				
Waters	220			1443	112
*R*_work_/*R*_free_ ¶ (%)	19.5/22.8			22.3/28.1	18.3/20.8
R.m.s. deviations††					
Bond lengths (Å)	0.005			0.012	0.005
Bond angles (°)	1.3			1.5	1.3

† *R*_merge_ = 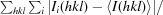 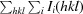, where *I* *_i_*(*hkl*) is the intensity of observed reflection *hkl* and 〈*I*(*hkl*)〉 is the mean intensity of symmetry-equivalent reflections.

‡ Data from *SHELXC* (Sheldrick, 2010 ▶).

§ Data from phasing program.

¶ *R*_work_ = 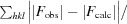 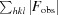. *R*_free_ was calculated using 5% of the reflections.

†† R.m.s. deviations in bond length and angles are the deviations from ideal values.

## Footnotes

1 Supplementary material has been deposited in the IUCr electronic archive (Reference: WD5183). Services for accessing this material are described at the back of the journal.
