# Supplementary material for: Experimental phasing using zinc anomalous scattering
Source: Acta Crystallogr D Biol Crystallogr. 2012 Aug 18;68(Pt 9):1253–8. doi: 10.1107/S0907444912024420 (PMC3489106; doi:10.1107/S0907444912024420)
Supplement: Supplementary file 1 [file d-68-01253-sup1.pdf]

Supplementary Material

PDB code	Crystallization condition		
1D1S<br>(Xie & Hurley, 1999)	0.1M Zinc Acetate<br>0.0075M NAD^+	0.1M Cacodylate pH 6.5	18% PEGL 6000
1FD9<br>(Riboldi-Tunnicliffe *et al.*, 2001)	0.5M Zinc Acetate	0.1M MES pH 6.1−6.5	15−20% PEG 8000
1K51<br>(O'Neill *et al.*, 2001)	0.225M Zinc Acetate	0.5M Cacodylate pH 6.5	2% PEG8000
1K52<br>(O'Neill *et al.*, 2001)	0.12M Zinc Acetate	0.5M Cacodylate pH 6.5	
1K53<br>(O'Neill *et al.*, 2001)	0.225M Zinc Acetate	0.5M Cacodylate pH6.5	
1NNK<br>(Lunn *et al.*, 2003)	0.1MZinc Acetate	0.1M Cacodylate pH 5.5	20% PEG 8000
1UV0 [NR]	0.2M Zinc Acetate	0.1M Cacodylate pH 6.0	20% PEG 8000<br>5% GLYCEROL
1UQW [NR]	0.2M Zinc Acetate	0.1M Sodium Acetate<br>pH 6.0	15% PEG 8000<br>5% GLYCEROL
1VPY [NR]	0.2M Zinc Acetate	0.1M Acetate pH4.5	10% PEG-3000
2AL4<br>(Jin *et al.*, 2005)	0.1-0.15M Zinc Acetate	0.1M Sodium Acetate<br>pH 5.5	10-14% PEG8000
2CH9<br>(Schuttelkopf *et al.*, 2006)	0.18M Zinc Acetate	0.18 M Acetic Acid<br>pH 4.6	9% PEG 3350
2GFE<br>(Weston *et al.*, 2006)	0.1M Zinc Acetate	0.1M Sodium Acetate<br>pH 5.5	14% PEG 8K
2HBB [NR]	0.2M Zinc Acetate<br>2.5M NaCl	0.01M Imidazole pH 8.0	
2IZO<br>(Dore *et al.*, 2006)	0.22M Zinc Acetate<br>0.03M GLYCYL-GLYCYL-GLYCINE	0.1M Acetate PH 4.8	8% PEG 8,000
2J6E<br>(Duquerroy *et al.*, 2007)	0.003M Zinc Acetate<br>0.003M CdCl2	0.1M CacodylatePH 6.5	10% MPEG 5000
2JGO<br>(Touw *et al.*, 2007)	0.2M Zinc Acetate	0.1M Imidazole pH 8.0	30% PEG-400
2W0M<br>(McRobbie *et al.*, 2009)	0.15M Zinc Acetate	0.1M MES PH 5.5,	PEG 8K 8.94%
2W9N<br>(Komander *et al.*, 2009)	0.2M Zinc Acetate<br>pH 7.0		22% PEG 3350
2XK5<br>(Virdee *et al.*, 2010)	0.2M Zinc Acetate<br>pH 7.0		19-20 % PEG 3350
2Y7I<br>(Stamp *et al.*, 2011)	0.2M Zinc Acetate	0.1M Cacodylate pH 7.4	18% PEG 8000
2ZCB [NR]	0.2M Zinc Acetate<br>pH 6.3		20% PEG 3350
3AAK<br>(Inuzuka *et al.*, 2010)	0.05M Zinc Acetate	0.1M Cacodylate pH 6.5	25% 2-methyl-2,4-pentanediol
3CJJ<br>(Koch *et al.*, 2010)	0.2M Zinc Acetate	0.1M Cacodylate pH 6.5	11% PEG 8000
3DP6<br>(Ahmed *et al.*, 2009)	0.1-0.2M Zinc Acetate	0.1M Cacodylate pH 6.5	14-17% PEG 8000
3DZA [NR]	0.2M Zinc Acetate<br>pH 6.3		20% PEG-3350
3EER [NR]	0.2M Zinc Acetate	0.1M Imidazole pH 8.0	20% PEG 3000
3FAV [NR]	0.2M Zinc Acetate	0.1M Imidazol pH 6.5	33% PEG 600
3FM4 [NR]	0.1M Zinc Acetate	0.1M Cacodylate pH 6.5	16% PEG 8000

3FJU<br>(Sanglas *et al.*, 2009)	0.2M Zinc Acetate	0.1M Cacodylate pH 6.5	9% PEG 8000
3H6T<br>(Hald *et al.*, 2009)	0.3M Zinc Acetate	0.1M Cacodylate pH 6.5	25 % PEG 4000
3IOT<br>(Kim *et al.*, 2009)	0.2M Zinc Acetate<br>0.2M Sodium Acetate	0.1M Cacodylate pH 6.5-7.4	12% PEG 4000
3IVB [NR]	50mM Zinc Acetate	0.1M Tris pH 7.6	10-20% PEG3350
3M3L<br>(Ahmed *et al.*, 2010)	0.1-0.15M Zinc Acetate<br>0.25M Ammonium Sulfate	0.1M Cacodylate pH 6.5	14-15% PEG 8000
3M52<br>(Stogios *et al.*, 2010)	0.075M Zinc Acetate<br>pH 4.6		
3M6Q<br>(Fieulaine *et al.*, 2011)	0.1M Zinc Acetate		20% PEG-3350
3MCX [NR]	0.2M Zinc Acetate	0.1M Cacodylate pH 6.5	2% 2-propanol
3OAX<br>(Makino *et al.*, 2010)	0.065 M Zinc Acetate<br>0.85 M Ammonium Sulfate	0.03 M MES pH 6.0	0.005 M □-mercaptoethanol
3P2X [NR]	0.04% Zinc Acetate	0.05M Sodium Citrate pH 8.3	1% phenol
3P33 [NR]	0.04% Zinc Acetate	0.05M Sodium Citrate pH 8.3	1% phenol
3R3Q<br>(Ren & Hurley, 2011)	0.2M Zinc Acetate	0.1M Imidazole pH 8.0	25% 1,2-propanediol<br>10% glycerol
3RF4<br>(Cho *et al.*, 2011)	0.16-0.24 M Zinc Acetate	0.1M Imidazole pH 8.0	15-25% PEG3000
3RTF<br>(Poon *et al.*, 2011)	0.1-0.15M Zinc Acetate<br>0.25M Ammonium Sulfate	0.1M SodiumCacodylate pH 6.5	14-15% PEG8000
3S9C<br>(Nakayama *et al.*, 2011)	0.2M Zinc Acetate<br>pH 6.0		20% PEG 3350

*NR: No reported primary citation

References

Ahmed, A. H., Ptak, C. P. & Oswald, R. E. (2010). *Biochemistry* **49**, 2843-2850.

Ahmed, A. H., Wang, Q., Sondermann, H. & Oswald, R. E. (2009). *Proteins* **75**, 628-637.

Cho, Y., Vermeire, J. J., Merkel, J. S., Leng, L., Du, X., Bucala, R., Cappello, M. & Lolis, E. (2011). *Chemistry & biology* **18**, 1089-1101.

Dore, A. S., Kilkenny, M. L., Jones, S. A., Oliver, A. W., Roe, S. M., Bell, S. D. & Pearl, L. H. (2006). *Nucleic acids research* **34**, 4515-4526.

Duquerroy, S., Stura, E. A., Bressanelli, S., Fabiane, S. M., Vaney, M. C., Beale, D., Hamon, M., Casali, P., Rey, F. A., Sutton, B. J. & Taussig, M. J. (2007). *Journal of molecular biology* **368**, 1321-1331.

Fieulaine, S., Boularot, A., Artaud, I., Desmadril, M., Dardel, F., Meinnel, T. & Giglione, C. (2011). *PLoS biology* **9**, e1001066.

Hald, H., Ahring, P. K., Timmermann, D. B., Liljefors, T., Gajhede, M. & Kastrup, J. S. (2009). *Journal of molecular biology* **391**, 906-917.

Inuzuka, T., Suzuki, H., Kawasaki, M., Shibata, H., Wakatsuki, S. & Maki, M. (2010). *BMC structural biology* **10**, 25.

Jin, R., Clark, S., Weeks, A. M., Dudman, J. T., Gouaux, E. & Partin, K. M. (2005). *The Journal of neuroscience : the official journal of the Society for Neuroscience* **25**, 9027-9036.

Kim, M. W., Chelliah, Y., Kim, S. W., Otwinowski, Z. & Bezprozvanny, I. (2009). *Structure* **17**, 1205-1212.

Koch, M., Chitayat, S., Dattilo, B. M., Schiefner, A., Diez, J., Chazin, W. J. & Fritz, G. (2010). *Structure* **18**, 1342-1352.

Komander, D., Reyes-Turcu, F., Licchesi, J. D., Odenwaelder, P., Wilkinson, K. D. & Barford, D. (2009). *EMBO reports* **10**, 466-473.

Lunn, M. L., Hogner, A., Stensbol, T. B., Gouaux, E., Egebjerg, J. & Kastrup, J. S. (2003). *Journal of medicinal chemistry* **46**, 872-875.
Makino, C. L., Riley, C. K., Looney, J., Crouch, R. K. & Okada, T. (2010). *Biophysical journal* **99**, 2366-2373.
McRobbie, A. M., Carter, L. G., Kerou, M., Liu, H., McMahon, S. A., Johnson, K. A., Oke, M., Naismith, J. H. & White, M. F. (2009). *Journal of molecular biology* **389**, 661-673.
Nakayama, D., Ben Ammar, Y., Miyata, T. & Takeda, S. (2011). *FEBS letters* **585**, 3020-3025.
O'Neill, J. W., Kim, D. E., Johnsen, K., Baker, D. & Zhang, K. Y. (2001). *Structure* **9**, 1017-1027.
Poon, K., Ahmed, A. H., Nowak, L. M. & Oswald, R. E. (2011). *Molecular pharmacology* **80**, 49-59.
Ren, X. & Hurley, J. H. (2011). *The EMBO journa* **30**, 2130-2139.
Riboldi-Tunnicliffe, A., Konig, B., Jessen, S., Weiss, M. S., Rahfeld, J., Hacker, J., Fischer, G. & Hilgenfeld, R. (2001). *Nature structural biology* **8**, 779-783.
Sanglas, L., Aviles, F. X., Huber, R., Gomis-Ruth, F. X. & Arolas, J. L. (2009). *Proceedings of the National Academy of Sciences of the United States of America* **106**, 1743-1747.
Schuttelkopf, A. W., Hamilton, G., Watts, C. & van Aalten, D. M. (2006). *The Journal of biological chemistry* **281**, 16570-16575.
Stamp, A. L., Owen, P., El Omari, K., Lockyer, M., Lamb, H. K., Charles, I. G., Hawkins, A. R. & Stammers, D. K. (2011). *Proteins* **79**, 2352-2357.
Stogios, P. J., Cuesta-Seijo, J. A., Chen, L., Pomroy, N. C. & Prive, G. G. (2010). *Journal of molecular biology* **400**, 983-997.
Touw, D. S., Nordman, C. E., Stuckey, J. A. & Pecoraro, V. L. (2007). *Proceedings of the National Academy of Sciences of the United States of America* **104**, 11969-11974.
Virdee, S., Ye, Y., Nguyen, D. P., Komander, D. & Chin, J. W. (2010). *Nature chemical biology* **6**, 750-757.
Weston, M. C., Gertler, C., Mayer, M. L. & Rosenmund, C. (2006). *The Journal of neuroscience : the official journal of the Society for Neuroscience* **26**, 7650-7658.
Xie, P. T. & Hurley, T. D. (1999). *Protein science : a publication of the Protein Society* **8**, 2639-2644.
